# Heavy Metal(oid)s Contamination and Potential Ecological Risk Assessment in Agricultural Soils

**DOI:** 10.3390/jox14020037

**Published:** 2024-05-14

**Authors:** Muhammad Saleem, David Pierce, Yuqiang Wang, Donald A. Sens, Seema Somji, Scott H. Garrett

**Affiliations:** 1 Department of Pathology, School of Medicine and Health Sciences, University of North Dakota, Grand Forks, ND 58202, USA; muhammad.saleem.1@und.edu (M.S.); donald.sens@und.edu (D.A.S.); seema.somji@und.edu (S.S.); 2 Department of Chemistry, University of North Dakota, Grand Forks, ND 58202, USA; david.pierce@und.edu (D.P.); yuqiang.wang@und.edu (Y.W.)

**Keywords:** agriculture soils, heavy metals, principal component analysis (PCA), enrichment factor (EF), ecological risk assessment

## Abstract

Soil pollution caused by heavy metal(oid)s has generated great concern worldwide due to their toxicity, persistence, and bio-accumulation properties. To assess the baseline data, the heavy metal(oid)s, including manganese (Mn), iron (Fe), Cobalt (Co), nickel (Ni), copper (Cu), zinc (Zn), arsenic (As), lead (Pb), mercury (Hg), chromium (Cr), and cadmium (Cd), were evaluated in surface soil samples collected from the farmlands of Grand Forks County, North Dakota. Samples were digested via acid mixture and analyzed via inductively coupled plasma mass spectrometry (ICP MS) analysis to assess the levels, ecological risks, and possible sources. The heavy metal(oid) median levels exhibited the following decreasing trend: Fe > Mn > Zn > Ni > Cr > Cu > Pb > Co > As > Cd > Hg. Principal component analysis (PCA) and hierarchical cluster analysis (HCA) suggested the main lithogenic source for the studied metal(oid)s. Metal(oid) levels in the current investigation, except Mn, are lower than most of the guideline values set by international agencies. The contamination factor (C_f_), geo accumulation index (I_geo_) and enrichment factor (EF) showed considerable contamination, moderate contamination, and significant enrichment, respectively, for As and Cd on median value basis. Ecological risk factor (E_r_) results exhibited low ecological risk for all studied metal(oid)s except Cd, which showed considerable ecological risk. The potential ecological risk index (PERI) levels indicated low ecological risk to considerable risk. Overall, the results indicate the accumulation of As and Cd in the study area. The high nutrients of the soils potentially affect their accumulation in crops and impact on consumers’ health. This drives the impetus for continued environmental monitoring programs.

## 1. Introduction

In addition to the significance of clean air and water, soil health is one of the key elements of environmental quality worldwide. A global problem endangering agricultural land and food security is the deterioration of soil quality. The environment is impacted by soil quality either directly or indirectly [1,2,3,4]. A crucial nonrenewable resource, soil functions as both the source and as a reservoir for numerous contaminants [5]. The contamination of agricultural soils by heavy metal(oid)s has drawn significant global attention due to their toxicity, persistent and non-biodegradable nature. In addition, human activities also substantially exacerbate heavy metal(oid) contamination [6,7,8,9]. In fact, heavy metal contamination contributes significantly to the deterioration of soil quality and environmental health [10,11,12]. 

Heavy metal(oid)s usually enter into the soils from lithogenic and anthropogenic sources [13]. Industrial activities, mining and smelting activities, electroplating, petrochemical activities, fossil fuel burning, waste disposal, agriculture activities, construction activities, irrigation water, vehicular exhausts, atmospheric deposition, etc., are potential anthropogenic sources of the heavy metal(oid)s in soils [12,14,15,16,17,18,19,20,21,22]. High levels of heavy metal(oid)s in soils are associated with a number of effects, such as changing the physicochemical properties of soil, affecting the human health (both directly and indirectly) and impacting animal life. Heavy metal(oid) concentration can also influence plants and impact crop quality and yield [14,23,24,25]. Therefore, the identification of sources and the quantification of heavy metal(oid)s in soil proves to be an effective approach for controlling and mitigation of inorganic pollutants, which is helpful for the formulation of suitable regulations for soil protection [16,26]. 

Heavy metal(oid)s in agricultural soil have the potential to enter the food chain and impact human health [27,28]. It is widely recognized that metals and metalloids are important components in numerous biological, chemical and molecular processes [29]. Trace metals such as Mn, Fe, Co, Cu and Zn are recognized as essential elements, but their elevated levels can become toxic. For example, Cu, an essential micronutrient, participates in cell wall metabolism, protein synthesis, hormone signaling, electron carrier proteins, e.g., plastocyanin [30,31,32], but in excess amounts is toxic to humans and causes health problems such as gastrointestinal disturbance, central nervous problems, liver and kidney damage, hepatic and renal damage, oxidative cell damage and cell death, etc. [33,34,35,36]. Metal(oid)s such as As, Cd, Hg, etc., do not have any role in the human body and are known as nonessential elements and are toxic at low levels [29,37]. An example is As, which causes cancer in humans; Cd can cause acute kidney injury, bone damage, and cancer in humans; Hg can damage the cardiovascular system, reproductive and immune systems, leading to premature death at high exposure levels; and Lead (Pb), a metabolic toxin affects the immune system, kidney, reproduction, and development at a low level [38,39,40,41,42,43]. 

Agricultural soil is important in producing various grains in the local area of Grand Forks County, and eastern North Dakota has one of the richest and fertile soils found in the USA, making agricultural endeavors one of the most successful economically. This success is underscored by the fact that at least 10 of the crops produced in North Dakota contribute to at least 30% or more of the total US production, and over 90% for canola and flax seed according to the USDA’s website. Glaciation contributes considerably to the geologic history of the local region, and the Red River valley resides in the sediment remnants of Lake Agassiz, a proglacial lake. A recent study indicates a natural high soil abundance of metals from the erosion and transport of Cretaceous shales [44]. Thus, the rich content of metallic nutrients exists in the context of many metals and metalloids, and understanding the dynamics that affect plant availability; the movement of these elements impacts local crop production, as well as human health, through the consumption of food products derived from these crops. There has been concern about the cadmium level and the accumulation of this metal in flax seed, for example [45]. Therefore, it is important to monitor the soil for heavy metal(oid) pollution for food safety. The bioavailability of metals and their toxicity to the biota depend on their chemical forms, which can be determined by a multi-step sequential extraction procedure, which provides acid extractable/exchangeable, reducible, oxidizable and residual/immobile fractions of metals in the soil. Although pseudo-total concentration does not indicate the bioavailability/mobility of the metal(oid)s, it provides the overall metal(oid) status in soils. There are several studies which were conducted worldwide to estimate metal contamination and associated risk assessment in agriculture soils [5,9,12,14,18,21]; however, there is very limited information on metal(oid) pollution available locally. In order to create baseline data of soil pollution, heavy metal(oid)s were analyzed for pseudo-total levels and the soil quality was assessed using various environmental quality indices. The main objectives of this study were (i) to assess the physicochemical properties (pH, EC, TDS) and pseudo-total levels of selected heavy metal(oid)s (Mn, Fe, Co, Ni, Cu, Zn, As, Pb, Hg, Cr, Cd) in soil samples; (ii) to compare the current metal(oid)s levels with guidelines set by international agencies and worldwide reported values; (iii) to assess the ecological risk by various indices i.e., geo–accumulation index (I_geo_), enrichment factor (EF), contamination factor (C_f_) and Ecological risk factor (E_r_) and Potential ecological risk index (PERI); and (iv) identify the possible sources of heavy metal(oid)s by multivariate analysis. Our long-term goal is to understand the distribution, geochemical fractionation for the bioavailability and speciation of metals, crop accumulation, the mobility of heavy metal(oid)s through this region and to what degree this impacts human health in future.

## 2. Material and Methods

### 2.1. Description of the Study Area

Grand Forks County, (48.0038° N, 97.3595° W) an agriculture county in the northeastern part of North Dakota, United States, is located in the Red River Valley region and covers a total area of approximately 1438 square miles (920,320 acres) with a population of 73,179. Grand Forks city is an important center of transportation, health care and education, with a miscellaneous collection of small industries. It has a sub-humid continental climate with great temperature variation during summer and winter. Ground water as well as surface water sources provide the main water supply in Grand Forks County. The soil has been formed by glacial activity and the accumulation of sediments by ancient Lake Agassiz, which is known for its rich and fertile soil. Few areas accumulate sufficient salts to form saline soils that reduce the productivity of over 80 k hectares, or 23% of the land area, in Grand Forks County. Soils containing soluble salts, most frequently sulfates and chlorides of calcium, magnesium, and/or sodium in sufficient amounts, are harmful to plants. Farming is the major enterprise in this area, and corn, soybeans, sunflowers, and spring wheat are the most common crops [46,47,48].

### 2.2. Sample Collection and Processing 

In September 2021, 15 representative soil samples (1–10 cm depth) were collected using a stainless-steel sampler by a random sampling technique from different agricultural land in Grand Forks County (48.0038° N, 97.3595° W), North Dakota, USA. Due to limited accessibility to all farmlands and landlords’ concerns, samples were not collected to cover all sides of the county, which would have been ideal. Each representative soil sample (almost 0.5–1 kg) was a combination of five to eight sub-samples which were taken around the same location (10–25 m^2^) and mixed thoroughly after carefully removing stones, gravel, and vegetation from the sampling site. To avoid cross-contamination, the sampler was cleaned after each sample with a Kimwipe to remove the soil particles, rinsed with deionized water, and wiped with a fresh Kimwipe. After sample collection in pre-cleaned zip-lock bags, the soil samples were transferred to the laboratory the same day. Samples were dried at 105 °C to remove water content. The samples were ground, homogenized, and sieved before storage for chemical analysis [49,50,51]. 

### 2.3. Quantification of Physicochemical Parameters and Heavy Metal(oid)s

For pH, electrical conductivity (EC) and total dissolved solids (TDS), as well as soil and water suspensions, were used [51,52]. For metal(oid) analysis, 0.5 g of dried powdered soil samples (<200 μm) was digested in a microwave with 4.5 mL conc. HNO_3_ and 1.5 mL conc. HCl [53]. The digested sample was centrifuged and filtered, and the volume was adjusted to 50 mL with deionized water. Further dilution was carried out before analysis if deemed necessary. In the present study, the samples were analyzed for Mn, Fe, Co, Ni, Cu, Zn, As, Pb, Hg, Cr and Cd using ICP MS (Thermo Scientific iCAP Qc) in kinetic energy discrimination mode (KED). All operating parameters were optimized using the manufacturer’s instructions to meet calibration and analysis requirements (Table S1). Mercury was analyzed with a Milestone DMA-80 Tri Cell direct mercury analyzer (Shelton, CT) (Table S2). All measurements were undertaken in triplicate and calibration line method. 

Quality control (QC) and quality assurance (QA) were assessed by reagent blanks, blank spikes, duplicate samples, and standard reference soils (NIST 2711a). The relative percent difference (RPD, %) of the heavy metal(oid) levels in the duplicate samples were less than 1–10% (Table S3). All reagents (acids, stock solutions and multi-element solutions) used in this study were of an analytical grade. Special attention was taken to reduce cross-contamination from air, glassware, and reagents in samples processing. The glassware were soaked with a 10% HNO_3_ solution overnight, then washed with deionized distilled water and dried prior to use in this study [54,55,56].

### 2.4. Soil Pollution Indices

#### 2.4.1. Geo-Accumulation Index (I_geo_)

The geo-accumulation index (I_geo_), commonly used for the pollution assessment of heavy metal(oid)s, is the ratio of the concentrations of heavy metal(oid)s in soils to geochemical background metal(oid)s levels in soils [56,57,58,59,60]. It is computed as follows:

(1) $$I_{geo}={log}_{2}(\frac{C_{n}}{{1.5B}_{n}})$$

where I_geo_, geo-accumulation index; C_n_, concentration measured of ‘n’ element in soils; B_n_, geochemical background value of the corresponding of ‘n’ element in the soil. The background reference value (mg/kg) for Mn, Fe, Co, Ni, Cu, Zn, As, Pb, Hg, Cr, Cd are 950, 56,300, 25, 84, 60, 70, 1.8, 14, 0.085, 102, 0.15, respectively [61]. The I_geo_ index classification about contamination level is given in Table 1. 

#### 2.4.2. Enrichment Factor (EF)

The EF is also another useful index to assess heavy metal(oid) pollution in soil and is given by the standardization of a measured metal(oid) against a reference metal. The referenced metals are Mn, Sc, Al, Fe, and Sn. In this study, we used Fe as a reference metal due to its relatively high levels in the earth’s crust [51,59,62,63,64].

(2) $$EF=\frac{{\left[X/M_{ref}\right]}_{sample}}{{\left[X/M_{ref}\right]}_{crust}}$$

where [X/M_ref_]_sample_ and [X/M_ref_]_crust_ refer to the ratio of mean concentrations (mg/kg) of the target metal(oid) in the examined soil and continental crust, respectively. The background reference values for EF calculation were used as given by Lide (2005) [61], and are interpreted by Sutherland (2000) [64] (Table 1).

#### 2.4.3. Contamination Factor (C_f_)

The contamination factor (C_f_) is the ratio between the metal(oid) levels whose contamination is being assessed and its preindustrial level is commonly found in the earth’s crust, and is computed as follows:

(3) Cf=CnCb

where ‘C_n_’ is the mean concentration of a metal(oid) in the soil and ‘C_b_’ refers to the earth crust/background value [59,61,63,65,66]. 

#### 2.4.4. Ecological Risk Factor (E_r_) and Potential Ecological Risk Index (PERI)

Ecological risk factor (E_r_) and Potential ecological risk index (PERI) were developed by Hakanson (1980) [66]. E_r_ allows for the assessment of each heavy metal(oid)’s ecological risk individually and is calculated as follows:

(4) $$E_{r}=T_{r}\times C_{f_{i}}$$

where Tr is the toxic response factor of heavy metal(oid)s (As = 10, Cd = 30, Cr = 2, Hg = 40, Cu = 5, Mn = 1; Ni = 5, Pb = 5, Zn = 1) and C_fi_ is the contamination factor of heavy metal(oid)s. The Potential ecological risk index (PERI) is a comprehensive method combining all of the heavy metal(oid)s’ toxicological effects and is measured through the following equation [51,56,59,63,65]:

(5) $$PERI=\sum_{i=1}^{n}E_{r}$$

Er and PERI classification is shown in Table 1.

### 2.5. Statistical Analysis

Minimum, maximum, mean, standard deviation (SD), Kurtosis, skewness, and the coefficient of variation (CV, %) of data was computed by MS Excel. Principal component analysis (PCA) and hierarchical cluster analysis (HCA) were carried out by SPSS software version 29. The Kolmogorov–Smirnov (K–S) test and the Shapiro–Wilk (S–W) test were used to measure the data normality. PCA and HCA were used to determine the relationships between the heavy metal(oid)s and their possible sources. The PCA validity was assessed by the Kaiser–Meyer–Olkin (KMO) value (KMO > 0.5) and Bartlett sphericity tests (*p* < 0.001) [67]. PCA is an exploratory data analysis technique that reduces the initial collection of highly correlated variables to a much smaller subset of uncorrelated variables known as principal components (PCs). Each component variance is exhibited by the eigenvalues, which were obtained by converting the original variables to PCs. Varimax with Kaiser Normalization was employed as the rotation method in PCA analysis [68,69]. HCA, a statistical tool, identifies clusters or groups based on their similarities in the data [20]. HCA was conducted using Ward’s method to assess the distances between two points [67,70,71], and cluster relationships between the heavy metal(oid)s were visually shown as a dendrogram.

## 3. Results and Discussion

### 3.1. Descriptive Statistics of Physiochemical Parameters and Heavy Metal(oid)s of Soil

The descriptive statistics analysis of physiochemical parameters (pH, EC, TDS) and heavy metal(oid)s (Mn, Fe, Co, Ni, Cu, Zn, As, Pb, Hg, Cr, Cd) are given in Table 2. The pH range (median in bracket) was noted to be between 6.6–7.1 (6.9), which indicates a slightly acidic to near-neutral soil nature. Such soil pH is optimal for plant growth. Generally, a pH range of 5.5 to 7.0 is considered optimal for most plants and vegetables [72]. The soil pH is important for the fertility of the soil, and it affects the nutrients availability to plants [73]. An acidic nature of soil enhances the mobility of heavy metal(oid)s in the soil while a slightly alkaline nature decreases the mobility of heavy metal(oid)s in the soil [5,74]. The soluble salt level in soil was assessed using electrical conductivity (EC) and total dissolved solids (TDS), which varied from 86.20–1883 µS/cm and 57.30–1251 mg/L with a median level of 305.5 µS/cm and 203.3 mg/L, respectively. There is currently no official guideline as to what is considered a safe level for TDS and EC, but elevated levels of TDS and EC are usually linked with higher levels of soluble ions. The heavy metal(oid) concentrations (mg/kg) varied between 664.7 and 1699 for Mn, 12,229 and 30,057 for Fe, 4.623 and 11.49 for Co, 15.07 and 36.83 for Ni, 7.614 and 25.49 for Cu, 45.60 and 89.25 for Zn, 3.592 and 17.66 for As, 6.379 and 14.77 for Pb, 0.019 and 0.104 for Hg, 15.21 and 28.76 for Cr, and 0.421 and 1.231 for Cd, with an median level of 1142, 16,332, 6.272, 20.37, 10.77, 64.00, 4.535, 8.687, 0.029, 19.01, and 0.500 mg/kg, respectively, showing a following decreasing trend on a median basis: Fe > Mn > Zn > Ni > Cr > Cu > Pb > Co > As > Cd > Hg. A Single Factor ANOVA of the metal data revealed that studied metal(oid) levels were noted to be significantly higher (F_ratio_ (154.6) > F_critical_ (1.910); *p* < 0.05). The standard deviation (SD) value reflects the heterogeneous distribution of metal(oid)s. High SD values showed high heterogeneous distribution. The SD results exhibited low heterogeneous distribution for all metal(oid)s except Mn and Fe in the investigated region. The normality of data was investigated using the Kolmogorov–Smirnov (K–S) test and the Shapiro–Wilk test (S–W) in SPSS software. The results indicate that Ni, Cu, As, Pb, Hg and Cd exhibited non-normal distribution, while Mn, Zn and Cr showed normal distribution, statistically. The asymmetry of physicochemical parameters and heavy metal(oid) distribution was determined by the skewness and kurtosis, which showed asymmetrical distribution with positive (right–handed) skewness and leptokurtic (peakedness). The metal(oid)s with a skew value between 1 and −1 showed normal distribution and more than one manifested abnormal distribution [21,75].

The coefficient of variation (CV) represents the degree of dispersion of the various variables in the data [76]. The CV of heavy metal(oid)s ranged from 19.03% for Cr to 70.68% for As, and showed the following decreasing trend: As > Hg > Cu > Cd > Mn > Fe > Co > Pb > Ni > Zn > Cr. According to Wilding (1985) [77], CV is categorized as high variation (CV > 36%), moderate variation (16 < CV ≤ 36%), and low variation (CV ≤ 16%) [78]. Thus, As had the largest CV (70.68%), followed by Hg (64.07%), Cu (41.33%) and Cd (41.14%), indicating high variation (CV > 36%), which suggested anthropogenic influences on these metal(oid) levels in the soil [79,80].

### 3.2. Comparison of Heavy Metal(oid)s in Soil with World-Wide Soil Guidelines and Reported Values

A comparison of the mean metal(oid) levels in oven-dried agriculture soil samples from the current study with world-wide soil guidelines is presented in Table 3. Heavy metal(oid) levels were compared with USEPA Ecological SSL (ESS), New York Background (NYB), Netherlands Soil Guidelines (NSG), Canadian Soil Quality Guidelines (CSG), Australia Ecological investigation levels (AEI), China Background Values (CBV), and Conterminous US data (CUS) guidelines. The data revealed that the Pb, Zn, Co and Hg mean levels were lower, and the Mn mean levels were found to be higher than ESS, AEI and CUS. The Cd mean level was greater than NYB and CBV; the As mean level was noted to be higher than NYB; the Ni level was found to be lower than ESS, NSG, CSG and AEI. Cr and Cu were only higher than NYB and CBV, respectively. Overall, metal(oid) levels in the current investigation, except Mn, are lower than most of the guidelines set by international agencies. 

A comparison of the heavy metal(oid) levels with relevant studies focusing on several countries is shown in Table 3. Almost all these studies used HNO_3_ and HCl for the pseudo-total concentrations of metal(oid)s in agriculture soils. Compared with the selected previous studies, mean levels of Cr, Cu, Co, Hg, and Ni were close to or lower than the reported values in different countries. The mean levels for As exceeded the reported levels for USA, Korea, Turkey and Malawi. Similarly, the Cd mean level was found to be higher than the reported levels of USA, Korea, Iran, Greece, Colombia, and Malawi. The Zn level was noted to be higher in USA, Korea, Turkey, Pakistan, Iran, and Malawi. Moreover, the Pb level was noted to be higher in Korea, Iran, Galápagos Islands, Colombia, and Malawi. Overall, Mn (100%), As (80%) Cd (55%), Zn (50%), and Pb (42%) were noted to be higher than the selected previous studies (Table 3). 

### 3.3. Source Identification Using Multivariate Analysis

Principal component analysis (PCA) and hierarchical cluster analysis (HCA) is a well-known statistical method, used to identify possible sources of heavy metal(oid)s in soil [67,98,99]. PCA was carried out when the KMO value was higher than 0.5 and *p* < 0.001 in Bartlett sphericity tests. The PCA results showed two principal components with 90.29% of cumulative variance. The PC 1 explained 77.29% of total variance and includes mostly strong loading of metal(oid)s, while PC 2 described 13.00% of the total variance, and includes moderate positive loading of Zn and Mn and strong negative loading of Hg. Similarly, HCA results exhibited three main clusters; (Fe-Ni-Co-Pb, Zn-Cr-Mn), (Cu-Cd-As), and Hg. The first cluster is further divided into two sub groups; 1a (Fe-Ni-Co-Pb) and 1b (Zn-Cr-Mn). Sub group 1a (Fe-Ni-Co-Pb) may be attributed to the lithogenic source. The sub group 1b and other two clusters (Zn-Cr-Mn, Cu-Cd-As, Hg) might be associated with lithogenic or anthropogenic sources. In multivariate analysis (PCA, HCA), Hg is often grouped as an isolated group [100]. Pollution indices results also suggested the contamination of As and Cd, followed by Mn and Zn. Arsenic, Hg, Cu and Cd showed higher CV. 

**Figure 1:**
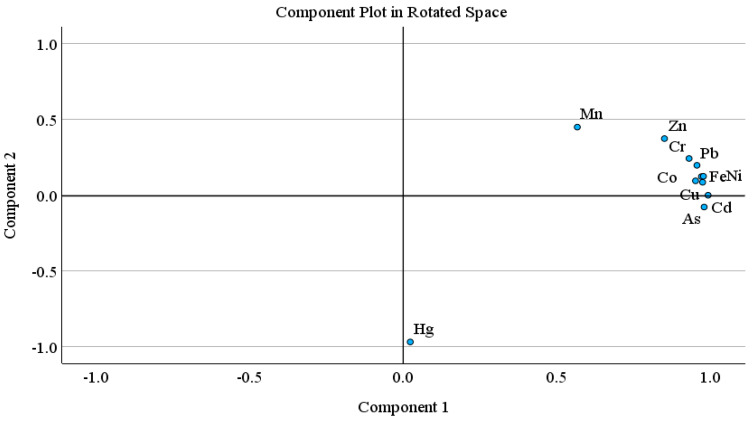
Principal component analysis loading plots for the two rotated components.

**Figure 2:**
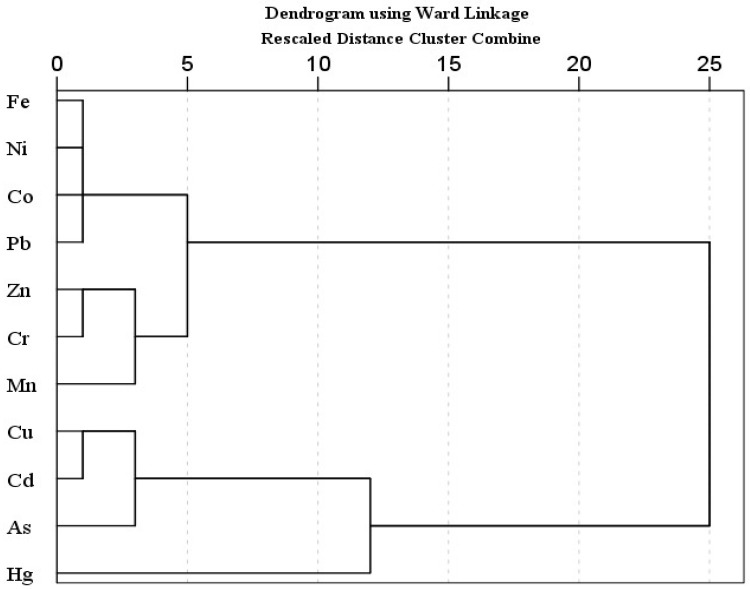
Hierarchical Cluster Analysis for heavy metal(oid) levels in soil samples.

Pollution indices, CV, and a comparatively higher level of Mn and Zn support the HCA clusters (Zn-Cr-Mn, Cu-Cd-As, Hg), suggesting the possible anthropogenic intrusion in agriculture soil as well. Agriculture activities (chemical fertilizers, pesticides or herbicides and transportation activities such as spraying, ploughing, and harvesting) and atmospheric deposition are the possible sources of metal(oid)s. According to previous studies, the Cd, Cu, As, Mn and Zn concentration in agriculture soils is correlated with agricultural activities, and atmospheric deposition [67,69,101,102,103,104]. Atmospheric deposition (traffic emission related to agricultural activities, coal and oil combustion, construction dust) is also one of the large sources of heavy metal(oid)s in farmland, other than fertilizer and pesticides [56,98]. Overall, the PCA results are in good agreement with the CA findings for studied metal(oid)s in the agriculture soil samples (Figure 1 and Figure 2).

### 3.4. Evaluation of Soil Pollution

#### 3.4.1. Geo–Accumulation Index (I_geo_)

**Figure 3:**
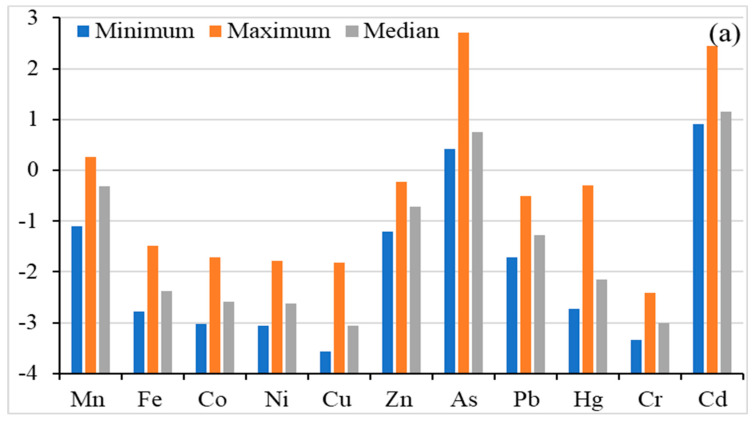

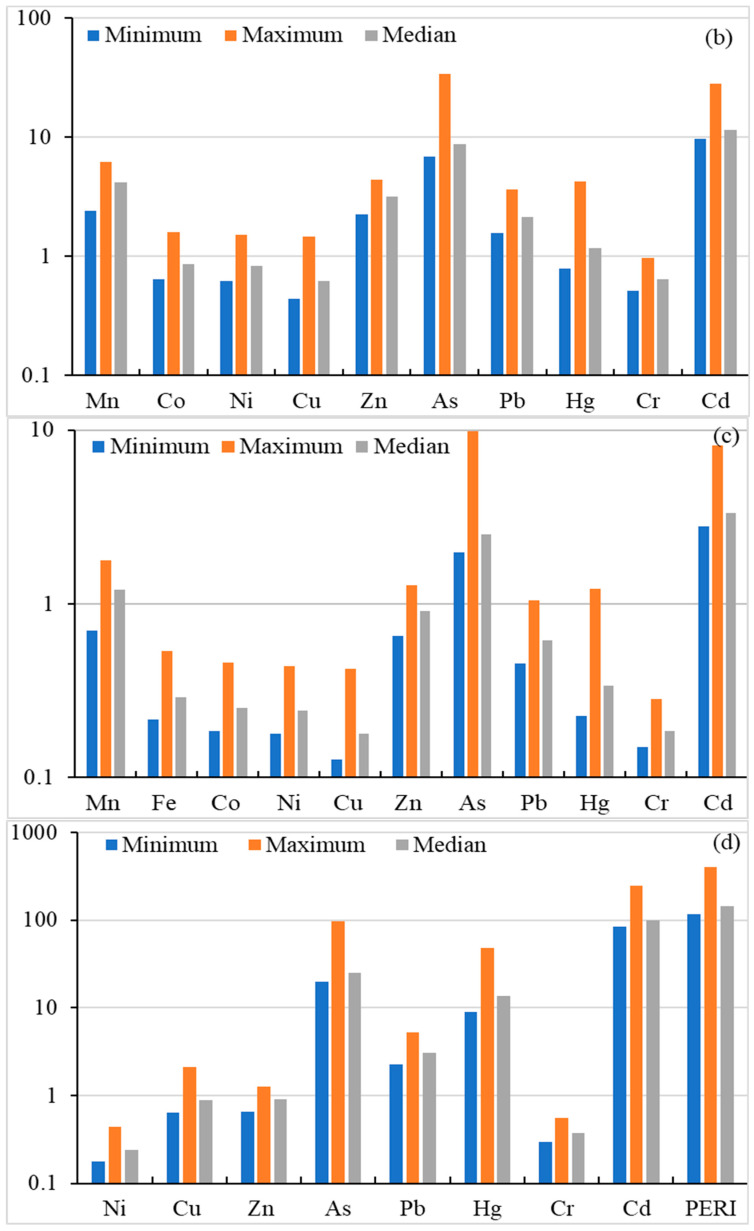
Risk assessment for heavy metal(oid)s; Geo-accumulation Factor (**a**), Enrichment Factor (**b**), Contamination factor (**c**), Ecological risk factor and Potential ecological risk index (**d**).

The geo-accumulation index (I_geo_) is used to measure the soil contamination induced by anthropogenic activities. Figure 3 shows the I_geo_ values of the studied heavy metal(oid)s in agricultural soil. The heavy metal(oid)s were found in the following decreasing order (I_geo_ calculated on median value): Cd (1.153) > As (0.748) > Mn (−0.319) > Zn (−0.714) > Pb (−1.274) > Hg (−2.148) > Fe (−2.370) > Co (−2.580) > Ni (−2.629) > Cu (−3.064) > Cr (−3.009), indicating uncontaminated to moderately contaminated for As and Cd, while the rest of the metal’s I_geo_ were below zero, indicating uncontaminated soils. I_geo_ values for Mn ranged from −1.100 to 0.254 which indicates uncontaminated to moderately contaminated soil. As ranged from 0.412 to 2.709 with a median value of 0.748, which shows two different degrees of contamination of As: uncontaminated to moderately contaminated and moderately to heavily contaminated. Similarly, Cd ranged from 0.904 to 2.452 with a median value of 1.153, indicating three different degrees of contamination: uncontaminated to moderately contaminated; moderately contaminated; moderately to heavily contaminated. For other metal(oid)s, except Cd, As and Mn, the maximum geo–accumulation index was found to be smaller than 0, which indicates that the agricultural soil is not contaminated with Fe, Co, Ni, Cu, Zn, Pb, Hg and Cr according to the Müller scale [57] and the only concern is with As and Cd. 

#### 3.4.2. Enrichment Factor (EF)

Enrichment factor (EF) is a commonly used parameter, to measure the level of contamination/enrichment of an element with respect to its background level in the Earth’s crust. Calculated EF values in agriculture soils are shown in Figure 3. The EF values (median value basis) of the studied metal(oid)s were found in the order of Cd (11.50) > As (8.684) > Mn (4.145) < Zn (3.152) > Pb (2.139) > Hg (1.167) > Co (0.865) > Ni (0.836) > Cr (0.642) > Cu (0.642), indicating minimal enrichment for Cu, Cr, Ni, Co and Hg. Manganese, Zn, and Pb showed moderate enrichment, while Cd and As indicated significant enrichment. In addition, the maximum EF values for As and Cd indicated a very high enrichment, Mn showed significant enrichment, and Zn, Hg and Pb showed moderate enrichment while rest of the metal(oid)s showed minimum enrichment, based on the classification given by Sutherland (2000) [64]. The results indicate that the soil in the study areas is contaminated with Cd and As, the main source of which is anthropogenic inputs from agriculture activities, as well as atmospheric deposition. Overall, the EF data in this study indicate no/minimal enrichment to significant enrichment in soil. 

#### 3.4.3. Contamination Factor (C_f_) 

Contamination factor (C_f_) is used to assess the soil contamination level and to infer anthropogenic intrusion. The calculated C_f_ values for heavy metal(oid)s are given in Figure 3. The C_f_ values of the studied metal(oid)s were found in the order of Cd (3.335) > As (2.519) > Mn (1.202) > Zn (0.914) > Pb (0.620) > Hg (0.339) > Fe (0.290) > Co (0.251) > Ni (0.242) > Cr (0.186) > Cu (0.179), indicating Cd has the highest C_f_ value while Cr has the lowest value. Cu, Cr, Ni, Co, Fe, Hg, Pb and Zn showed CF < 1.0, indicating low contamination of the studied soils. The median based C_f_ value for As and Mn showed moderate contamination, whereas the C_f_ value Cd indicated considerable contamination. On the basis of the maximum C_f_ value, Pb, Hg, Zn and Mn indicated moderate contamination while As and Cd indicated a very high contamination level. Overall C_f_ indicates moderate to considerable contamination for Mn, As and Cd in studied soils. 

#### 3.4.4. Ecological Risk Factor (E_r_) and Potential Ecological Risk Index (PERI)

The ecological risk factor was used to assess the sensitivity of several biological communities to toxic metal(oid)s. In the current investigation, E_r_ and PERI values are calculated and presented in Figure 3. The E_r_ level based on median level of Ni, Cu, Zn, As, Pb, Hg, Cr, Cd were found as 0.242, 0.897, 0.914, 25.19, 3.102, 13.54, 0.373 and 100.1, respectively, and exhibited the following decreasing order: Cd > As > Hg > Pb > Zn > Cu > Cr > Ni. The E_r_ results indicate low ecological risk for all metal(oid)s except Cd, which showed considerable ecological risk. On a maximum E_r_ value basis, Hg showed moderate ecological risk, As exhibited considerable ecological risk, while Cd posed a high ecological risk. The PERI levels were calculated as the sum of ecological risk (E_r_) of metal(oid)s. The PERI values ranged from 117.3 to 402.9, with a median value of 144.3, indicating low ecological risk to considerable risk. 

## 4. Conclusions

Soil is a major pool for contaminants and the heavy metal(oid) contamination of agricultural soil has become a severe environmental issue and a potential threat to food safety worldwide. This study provides valuable data about heavy metal(oid) levels in agriculture soils. The present study assessed the heavy metal(oid) levels, identifying a possible source as well as an ecological risk assessment (I_geo_, EF, C_f_, E_r_, and PERI) in agriculture soil in Grand Forks County, ND. The pH indicated slightly acidic to neutral soil nature, which is optimal for plant growth. The heavy metal(oid) concentration showed the following decreasing trend (median basis): Fe > Mn > Zn > Ni > Cr > Cu > Pb > Co > As > Cd > Hg. The Kolmogorov–Smirnov (K–S) test and the Shapiro–Wilk test (S–W) indicated that Ni, Cu, As, Pb, Hg and Cd were not normally distributed (*p* < 0.05). All the metal levels in the current investigation, except Mn, are lower than most of the guidelines set by international agencies, while Mn (100%), As (80%) Cd (55%), Zn (50%), and Pb (42%) were noted to be higher than the selected previous studies. Principal component analysis (PCA) and hierarchical cluster analysis (HCA) indicated a lithogenic source mainly of the studied metal(oid)s, while the presence of Hg, As, Cd, Mn and Zn might be attributed to anthropogenic sources as well. The I_geo_ values (median based) showed uncontaminated to moderate contamination for As and Cd, while the EF values of the studied metal(oid)s indicated significant enrichment for Cd and As. The C_f_ value for Mn and As showed moderate contamination, whereas the Cf value Cd indicated considerable contamination. E_r_ results indicate low ecological risk for all metal(oid)s except Cd, which showed considerable ecological risk. The PERI levels indicated low ecological risk to considerable risk. Overall, pollution indices indicated that the study area is contaminated with Cd and As, mainly, and should be monitored on a regular basis in the future. 

## Figures and Tables

**Table 1:** Description of geo-accumulation index (I_geo_), enrichment factor (EF), contamination factor (C_f_), Ecological risk factor (Er) and Potential ecological risk index (PERI) in soil.

Value	Sediments Quality	Value	Sediments Quality
I_geo_ ≤ 0	practically uncontaminated	E_r_ < 40	low risk
0 < I_geo_ < 1	uncontaminated to moderately contaminated	40 ≤ E_r_ < 80	moderate risk
1 < I_geo_ < 2	moderately contaminated	80 ≤ E_r_ < 160	considerable risk
2 < I_geo_ < 3	moderately to heavily contaminated	160 ≤ E_r_ < 320	high risk
3 < I_geo_ < 4	heavily contaminated	E_r_ ≥ 320	very high risk
4 < I_geo_ < 5	heavily to extremely contaminated		
5 < I_geo_	extremely contaminated		
EF < 2	minimal enrichment	PERI < 150	low risk
2 ≤ EF < 5	moderate enrichment	150 ≤ PERI < 300	moderate risk
5 ≤ EF < 20	significant enrichment	300 ≤ PERI < 600	considerable risk
20 ≤ EF < 40	very high enrichment	PERI ≥ 600	very high risk
EF ≥ 40	extremely high enrichment		
C_f_ < 1	low contamination		
1 ≤ C_f_ < 3	moderate contamination		
3 ≤ C_f_ < 6	considerable contamination		
6 ≤ C_f_	very high contamination		

**Table 2:** Descriptive data of physicochemical parameters and heavy metal(oid)s (mg/kg) in agriculture soil.

	Mn	Fe	Co	Ni	Cu	Zn	As	Pb	Hg	Cr	Cd	pH	EC	TDS
Minimum	664.7	12229	4.623	15.07	7.614	45.60	3.592	6.379	0.019	15.21	0.421	6.6	86.20	57.30
Maximum	1699	30057	11.49	36.83	25.49	89.25	17.66	14.77	0.104	28.76	1.231	7.1	1883	1251
Median	1142	16332	6.272	20.37	10.77	64.00	4.535	8.687	0.029	19.01	0.500	6.9	305.5	203.3
Kurtosis	−0.739	3.612	4.660	4.228	3.550	−0.118	3.796	1.524	6.976	0.802	3.34	0.343	1.155	1.129
Skewness	0.386	1.737	1.892	1.921	1.929	0.452	2.185	1.458	2.565	1.022	2.03	−0.544	1.421	1.416
CV	27.90	27.64	26.50	25.51	41.33	19.58	70.68	26.37	64.07	19.03	41.14	2.192	103.9	103.8
K-S test	0.200	0.135	0.077	0.004	0.004	0.200	<0.001	0.002	0.001	0.126	<0.001	0.200	0.048	0.048
S-W test	0.351	0.029	0.021	0.004	0.003	0.722	<0.001	0.010	<0.001	0.237	<0.001	0.218	0.009	0.009

**Table 3:** Mean metal concentrations (mg/kg) in the soil in comparison with the international guideline values and the worldwide reported levels.

	Mn	Co	Ni	Cu	Zn	As	Pb	Hg	Cr	Cd	
Current Study	1191	6.709	22.05	12.39	66.39	6.302	9.454	0.037	20.39	0.606	Present study
USEPA Ecological SSL	220	13	38	70	160	18	120	-	-	32	USEPA 2005 [81]
New York Back ground	-	-	17	14	65	5	19	-	14	0.5	Cheng et al., 2015 [82]
Netherlands Soil Guidelines	-	9	35	36	140	29	85	0.3	100	0.8	RIVM, 2001. [83]
Canadian Soil Quality Guidelines	-	40	45	63	250	12	70	6.6	64	1.4	CCME [84]
Australia Ecological investigation levels	500	50	60	100	200	20	600	1	400	3	Abraham et l., 2018 [85]
China Background Values	-	-	9.6	10.5	36.3	6.8	29.8	0.055	35.6	0.041	Cai et al., 2019 [86]
Conterminous US data	487	10	20	25	63	6.8	22	-	54	-	Goldhaber et al., 2009 [87]
	Worldwide reported values										
USA	629	-	16.9	12.6	55.2	6.25	14.9	-	26.2	0.3	Zhang, 2018 [88]
Korea	-	-	8.24	14.82	41.10	4.80	7.70	0.03	-	0.14	Kim et al., 2020 [89]
Turkey	-	-	85.02	43.19	65.10	5.66	17.01	-	194.7	-	Baltas et al., 2020 [90]
Pakistan	399.0	12.45	30.67	16.02	39.14	-	15.83	-	30.59	0.768	Batool Shah, 2023 [91]
Iran	561.8	15.1	109.3	23.75	56.6	-	8.31	0.13	67.3	0.32	Bahrami et al., 2019 [92]
Greece	1020	21.99	146.8	74.68	74.88	6.95	19.74	-	83.12	0.54	Kelepertzis, 2014 [19]
Morocco	-	-	-	138.1	162.1	-	31.72	-	32.72	0.92	Ennaji et al., 2020 [59]
Galápagos Islands	-	37.3	29.5	109	226	-	3.08	-	67.6	0.942	Dinter et al., 2021 [93]
Kosovo	-	-	156.5	33.35	90	-	163.3	-	92.3	1.005	Zogaj et al., 2014 [94]
Colombia	-	-	587	1004	1218	-	0.066	0.177	-	0.035	Marrugo et al., 2017 [95]
Herzegovina	-	31.42	34.53	44.20	97.03	-	44.30	-	-	0.76	ŠUKALIĆ et al., 2018 [96]
Malawi	-	-	16.32	13.45	36.71	1.09	6.54	-	26.77	BDL	Mussa et al., 2020 [97]

## Data Availability

Data are contained within article.

## Supplementary Materials

The following supporting information can be downloaded at: https://www.mdpi.com/article/10.3390/jox14020037/s1, Table S1: Instrumental operating parameters for ICP MS (iCAP, Thermo Fisher) for the selected metal(oid)s analysis; Table S2: Instrumental operating parameters for Direct Mercury Analyzer (Milestone DMA-80 Hg analyzer) for the Mercury analysis; Table S3: Limit of Detection, Limit of Quantitation (µg/kg), Method Blank, SRM (2711a) recovery (%) Blank spike recovery (%) and relative percent difference (RPD, %) of duplicate sample analysis for the selected metal(oid)s analysis.
