# Supplementary material for: Heavy Metal(oid)s Contamination and Potential Ecological Risk Assessment in Agricultural Soils
Source: J Xenobiot. 2024 May 14;14(2):634–50. doi: 10.3390/jox14020037 (PMC11130943; doi:10.3390/jox14020037)
Supplement: Supplementary file 1 [file jox-14-00037-s001.zip › jox-2941067-supplementary.pdf]

Journal of Xenobiotics

MDPI

Supplementary Materials: Heavy Metal(oid)s Contamination and Potential Ecological Risk Assessment in Agricultural Soils

Muhammad Saleem, David Pierce, Yuqiang Wang, Donald A. Sens, Seema Somji and Scott H. Garrett

Table S1. Instrumental operating parameters for ICP MS (iCAP, Thermo Fisher) for the selected metal(oid)s analysis.

Plasma gas flow rate	14.0 L/min
Auxiliary gas flow rate	0.8 L/min
Carrier gas flow rate	0.8 L/min
RF power	1550 W
Nebulizer gas flow	1.06 L/min
Make up gas flow	0.25 L/min
Dwell time	0.05 sec
Analysis mode	KED
Nebulizer	1.03 L/min
Nebulizer	Microflow PFA-ST
Spray chamber	Peltier-cooled quartz cyclonic spray chamber
Spray chamber Temperature	2.7 °C
Sampling depth	5.0 mm
Sample uptake rate	0.4 mL/min
Sampler/Skimmer Cone	Nickel
Detector mode	Auto
Number of replicates	3
Sweeps	10

Table S2. Instrumental operating parameters for Direct Mercury Analyzer (Milestone DMA-80 Hg analyzer) for the Mercury analysis .

Carrier gas	High Purity Oxygen
Furnace 1, drying	70 s at 250 °C
Furnace 1, decomposition	180 s at 650 °C
Furnace 2, catalyst	565 °C
Amalgamator heating temperature	850 °C
Cuvette temperature	120 °C
Maximum start Temperature	250 °C
amalgamator standby temperature	170 °C
Purge time P	60 s
amalgamator Heater time	12 s
Signal recording time R	30 s
O_2 Flow rate	7 L/h

Table S3. Limit of Detection, Limit of Quantitation (μg/kg), Method Blank, SRM (2711a) recovery (%) Blank spike recovery (%) and relative percent difference (RPD, %) of duplicate sample analysis for the selected metal(oid)s analysis.

Metal	LOD	LOQ	Method Blank	SRM Recovery (%)	Blank Spike Recovery (%)	Relative Percent Difference (%)
Mn	0.135	0.431	ND	94.40%	119.0%	6.996%
Fe	2.307	7.349	ND	99.11%	137.3%	7.395%
Co	0.043	0.137	ND	85.96%	114.2%	1.791%
Ni	0.516	1.643	ND	94.53%	115.6%	3.716%
Cu	0.141	0.450	ND	97.76%	119.7%	0.333%
Zn	1.372	4.370	ND	103.2%	113.5%	3.248%
As	0.054	0.171	ND	105.6%	105.7%	10.44%
Cr	0.823	2.621	ND	63.23%	108.0%	2.527%
Pb	0.050	0.159	Detected	108.7%	107.0%	3.635%
Cd	0.023	0.073	ND	117.6%	110.1%	5.950%

ND; Not Detected (value < LOD); Detected, (LOD < value < LOQ).
